# Letter to the Editor: Knowledge gap in assessing the risk of a human pandemic via mammals’ infection with highly pathogenic avian influenza A(H5N1)

**DOI:** 10.2807/1560-7917.ES.2023.28.9.2300134

**Published:** 2023-03-02

**Authors:** Hiroshi Nishiura, Taishi Kayano, Katsuma Hayashi, Tetsuro Kobayashi, Yuta Okada

**Affiliations:** 1 School of Public Health and Graduate School of Medicine, Kyoto University, Kyoto, Japan

**To the Editor:** Following the infection event of highly pathogenic avian influenza (HPAI) A(H5N1) in farmed minks [1], poultry farms were also reported as affected by the same virus in Spain from October 2022 [2]. The virus contains a second sialic acid binding site in neuraminidase [3]. In addition to these reports, infection events in otters in England and sea lions in Peru brought people’s attention to infection in mammals and possible increased fitness of the virus in mammals. Nevertheless, it has never been systematically clarified how these reports inform the quantitative risk assessment for humans. Here we would like to discuss critical knowledge gaps in this area.

To our knowledge, it is the first time that outbreaks of HPAI A(H5N1) have occurred within the same season not only in wild birds and poultry but also in several mammalian species in various geographical locations. As Aznar et al. noted, it is unusual for bird-to-human transmission to occur frequently [2], and person-to-person transmission of HPAI A(H5N1) has been limited [4]. In light of these conventional understandings, we may have to focus on two possible pathways that could potentially lead to a pandemic in humans, i.e. (i) the risk of a human pandemic directly emerging from infected mammals and (ii) the risk of a human pandemic caused by a reassorted virus from pigs that acquired infection from other mammals. 

As for the former, it can be assumed that human exposure to minks and otters/sea lions is limited in the general population, and as long as the transmission remains restricted to those mammals, only emergence via occupational contact within farms and aquariums might be a concern. In addition, this epidemic season suggests that we need to understand the frequency of human exposure to different animal species, possibly across the world (e.g. as done in Nguyen et al. [5]). In high-income countries, perhaps infections in chicken have been of greater concern for humans than those in minks or otters, and exposure to companion animals including dogs and cats may be most frequent, followed by cattle, wild pigs and deer. We would need to enumerate even other animal species with less frequent contact, and the importance of farmed minks and otters should be quantitatively assessed in the context of the probability (risk) of animal-to-human transmission. 

Even less is known about the second risk, i.e. emergence of reassorted virus after mammal-to-swine transmission. It is well understood that knowing the frequency of pigs exposed to other animals is of importance in estimating the risk of reassortment [6]. Now also the regularity and closeness of contact between various mammals and pigs should be assessed so that the frequency of virus transmissions between them can be quantified.

In addition to characterising cross-species contact, the host range of circulating HPAI A(H5N1) should be investigated. Genomic characterisation of cross-species transmission may be established by comparing ongoing viruses against pre-existing HPAI A(H5N1). Not only cross-species barriers but also the capacity to cause mammal-to-mammal transmission plays a key role in pandemic emergence, and this increased transmissibility could also be attributed to viral genomic evolution as demonstrated by experimental study [7]. The global sharing of such information coupled with a multidisciplinary approach can accelerate the monitoring of pandemic emergence. We propose to systematically and routinely conduct such risk assessment to monitor the pandemic potential of HPAI A(N5N1).
